# Effectiveness of the application of a digitalized repository on the quality of graduate and post-graduate education in the field of physical medicine and rehabilitation

**DOI:** 10.3389/fmed.2024.1521959

**Published:** 2025-01-07

**Authors:** Ivet B. Koleva, Borislav R. Yoshinov, Radoslav R. Yoshinov

**Affiliations:** ^1^Medical University of Sofia Physiotherapy Department, Sofia, Bulgaria; ^2^Cardiorehabilitation Department, National Heart Hospital, Sofia, Bulgaria; ^3^Physical Medicine Department, Hospital for Longtime Care and Rehabilitation “Serdika”, Sofia, Bulgaria; ^4^Neurology Department, University Hospital for Active Care “St Ivan Rilsky”, Sofia, Bulgaria; ^5^University of Library Studies and Information Technologies, Sofia, Bulgaria

**Keywords:** digital repository, electronic library, education, physical medicine, physiatry, physical therapy, rehabilitation

## Abstract

**Background:**

During last ten years, we have developed a digital library with educational materials in Physical medicine and rehabilitation.

**Objectives:**

The objective of current article is the preparation of an electronic library with educational materials in the area of physical medicine, physical therapy and rehabilitation, and the comparative evaluation of the impact of this repository on the quality of education of students and trainees in the field.

**Methodology:**

The electronic library includes e-books on different topics, elements of the specialty “Physical and rehabilitation medicine (PRM)” or Physiatry – with theoretical data, practical issues and case reports with videos of real patients. From the field of basic physiatry we included: books on kinesiological and pathokinesiological analysis, functional evaluation; physiotherapy (active exercises, soft-tissue techniques), ergotherapy (activities), balneotherapy (mineral baths and peloids, underwater gymnastics), manual therapy (tractions, mobilizations, manipulations), preformed physical factors (electric currents, magnetic field, ultrasound, light, laser, etc). In special physiatry we prepared manuals on: neurological and neurosurgical rehabilitation, orthopedic and traumatologic rehabilitation, rheumatologic rehabilitation, cardiac and cardiosurgical rehabilitation, etc. The digital library has been applied to educate 1,186 learners (graduate and post-graduate studies): students in Bachelor’s degree (Physiotherapy, Nursing, Midwifery, Medical assistants) and in Master’s degree (Physiotherapy, Medical rehabilitation and balneology), medical doctors—residents in Physical and Rehabilitation medicine (PRM), participants in long-life learning courses (physiatrists, physiotherapists and nurses). At the end of every educational course, we collected data from the practical and theoretical exams (investigation period—from April 2020 to October 2024). Additionally, we evaluated learners’ opinion using anonymous questionnaires on the usefulness of the electronic library.

**Results:**

Comparative analysis of the results revealed significant improvement in theoretical knowledge and practical skills of students and trainees, as evidenced by online assessments, and face-to-face theoretical and practical exams. The majority of learners consider the electronic repository useful.

**Conclusion and recommendations:**

The authors consider this electronic library (with books, manuals, video-presentations and case studies) a valuable resource in both graduate and post-graduate education within the rehabilitation field. We recommend the application of electronic repositories in the process of education of the students and trainees.

## 1 Introduction

The accelerated development of medical science and practice necessitates rapid adaptation of education, to incorporate new advances. Educational strategies in the fields of Medicine, Public health and Healthcare are crucial for optimizing learner success and improving the quality of care in clinical practice.

### 1.1 Electronic learning

The European Center for the Development of Professional Education and Training defines electronic learning (e-learning) as an “instructional model, based on the use of information and communication technologies (ICT), to acquire knowledge, know-how, information, values, skills and competences through the Internet” (1). E-learning encompasses various formats and hybrid methodologies, such as programming systems, the Internet, CD-ROMs, real-time education via personal computers (PCs), and other electronic or interactive devices.

E-learning has become integral part of science and practice, including in medicine and health care, and education in these fields (2). During the COVID-19 pandemic, digitalization of educational materials became essential, and electronic education was widely adopted in medical universities. Therefore, over recent years, we have prepared a digital library of educational materials in the field of Physical Medicine and Rehabilitation.

### 1.2 Medical and healthcare education

The Council of the European Union of Medical Specialists defines the Medical Act as follows: “The medical act encompasses all the professional actions, e.g., scientific, teaching, training and educational, clinical and medico-technical steps, performed to promote health and functioning, prevent diseases, provide diagnostic or therapeutic and rehabilitative care to patients, individuals, groups or communities in the framework of the respect of ethical and deontological values. It is the responsibility of, and must always be performed by a registered medical doctor/physician or under his or her direct supervision and/or prescription” (3).

The quality of medical and healthcare services is essential, and continuous education is necessary. To support high standards of medical care, the European Union of Medical Specialists (UEMS) has developed normative documents for specialization of medical doctors and for Long-life learning (LLL) in the European Community: standards for promotion of good medical care (4), training medical specialists (5), continuing medical education (6), etc.

### 1.3 Physical medicine and rehabilitation

Physical and Rehabilitation Medicine (PRM), also known as physiatry, is a medical specialty that utilizes various physical modalities to improve the quality of care and patients’ quality of life. According to the European Union of Medical Specialists – PRM Section, it is an „independent clinical medical specialty, oriented to the promotion of physical and cognitive functioning, activities, participation and changes in personal factors and environment” (7). Medical doctors – PRM specialists are responsible for the management of the prevention, diagnostics, treatment and rehabilitation of patients with health-related disability and co-morbidity of all ages (7, 8).

The White Book on Physical and Rehabilitation Medicine defines the primary objective of PRM as optimizing social participation and enhancing patients’ quality of life (8). This includes supporting patient to achieve possible levels and patterns of autonomy and independence, including participation in professional, social and leisure activities, which are part of their human rights. Tasks of PRM are as follows: treating existing pathology; reducing disability; preventing and managing complications; enhancing functioning and activity; and encouraging patient’s participation in various activities (8, 9).

Rehabilitation is a functional therapy, based on detailed functional assessment. The World Report on Disability outlines rehabilitation goals as follows: preventing loss of function; slowing the rate of loss of function; improving or restoring function; compensating the lost function; and maintaining current function (10). Modern rehabilitation adopts an integrative and holistic approach to patient care, based on the International Classification of Diseases (ICD) (11); the International Classification of Functioning, disability and Health (ICF) (12), and clinical principles (13).

In clinical rehabilitation practice, we apply a complex algorithm, including a detailed functional evaluation and a synergic combination of different natural and preformed physical modalities (the “rehabilitation puzzle”). Natural physical factors include procedures of physical therapy (active and passive movements); hydrotherapy, balneotherapy and thalassotherapy (baths and pools with water, mineral and seawaters; jets; underwater exercises and underwater massage); climatotherapy; occupational therapy or ergotherapy (activities of daily life, professional activities). From the group of preformed modalities, we apply procedures of electrotherapy (electric currents and electrostatic field), magnetotherapy (magnetic field), ultrasound, photo-therapy (infrared, visible or ultraviolet beams of light, laser), among others.

**Figure 1:**
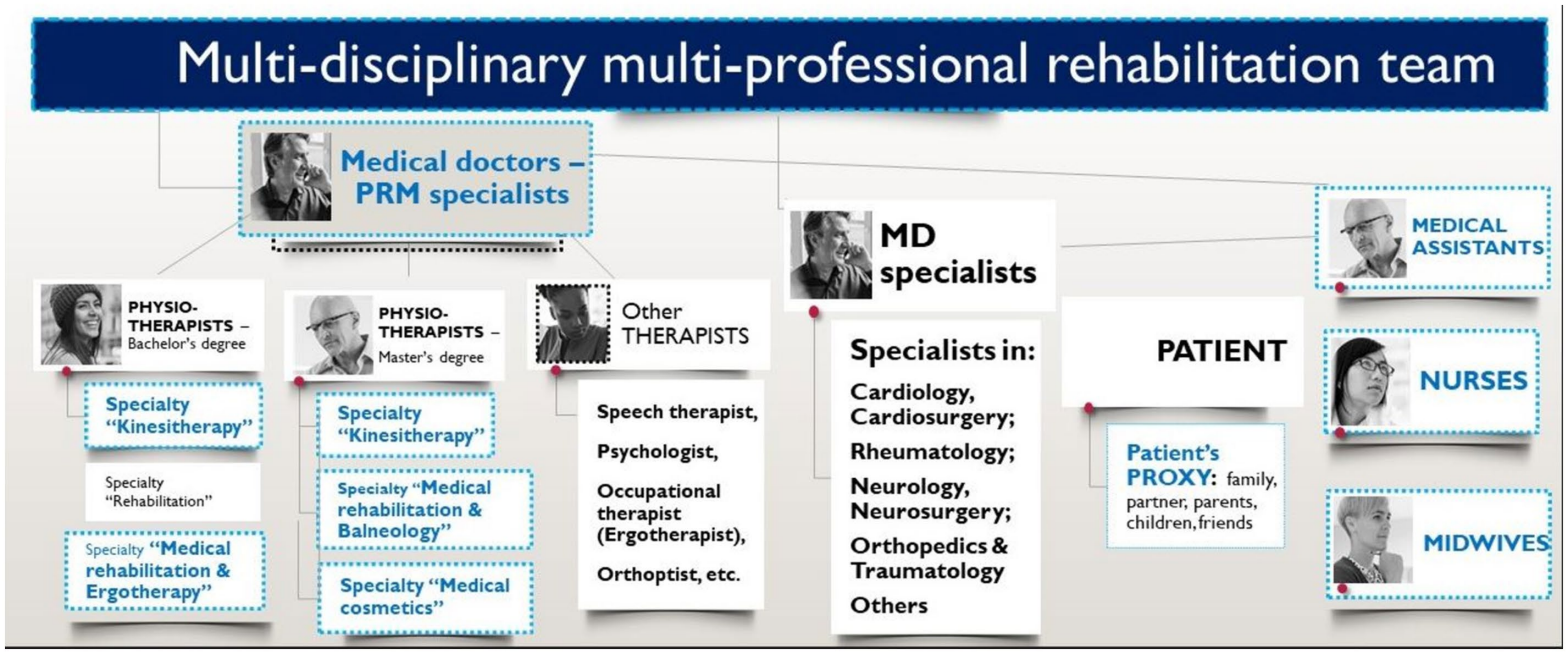
Multi-disciplinary and multi-professional rehabilitation team.

The World Report on Disability of the World Health Organization (WHO) and of the World Bank categorizes rehabilitation measures into three categories: rehabilitation medicine, therapy and assistive technologies (9). The rehabilitation process is carried out by a multidisciplinary and multiprofessional rehabilitation team, which includes medical doctors of various specialties, nurses, physiotherapists, occupational therapists, etc. Figure 1 presents the members of this rehabilitation team, with certain members highlighted in color to indicate their inclusion in our study.

### 1.4 Electronic learning in the rehabilitation practice

We adapt the educational process to the needs and expectations of different members of the rehabilitation team: medical doctors, physiotherapists, nurses, medical assistants, and midwives – during the process of their graduate and post-graduate education (as students, residents, and participants in long-life learning courses).

We consider basic principles of learning and e-learning (2, 14–16). We use the ABCD-model for learning, which emphasizes education, adapted to the audience (A) and its behavior (B), oriented toward the condition (C) and the degree (D). At every stage, we inform learners of our expectations, regarding their theoretical knowledge and practical skills. We provide clear instructions for education and vocational training, and we encourage the practical application of theoretical knowledge.

In clinical rehabilitation practice and in the process of education and training, we apply the SMART model: setting goals, which are specific (S), measurable (M) and attainable (A); with relevant (R) results and a time-bound (T) approach.

We have systematically introduced elements of electronic (e-) learning into the clinical practice of various educational disciplines – in the areas of basic principles of physical medicine and specialized rehabilitation (17–19). In the general section, we included topics such as: kinesiological analysis, manual therapy, balneology, thalassotherapy, ergotherapy (occupational therapy), and grasp and gait rehabilitation. In the clinical section, we covered: neurological and neurosurgical rehabilitation, pediatric neurorehabilitation, cardiorehabilitation, physical prevention, prevention and rehabilitation in Obstetrics and Gynecology, rehabilitation for SARS-CoV2 and Neuro-COVID, rehabilitation for PC-induced health issues, burnout prevention and rehabilitation, rehabilitation using neurorobots and virtual reality, and more. Current article summarizes the results of implementing electronic learning in all areas of PRM and evaluates its effectiveness and impact on the professional competencies of different types of the rehabilitation staff.

## 2 Aim and hypothesis

### 2.1 Goal

The objective of current article is to prepare an electronic library with educational materials in the field of physical medicine, physical therapy and rehabilitation, and to evaluate comparatively the impact of including this digitalized repository in the education of students and trainees in the field – on their professional competencies (theoretical knowledge and practical skills).

### 2.2 Hypothesis

The null hypothesis (H0) is that the application of elements of electronic learning will not influence the quality of education of members of the rehabilitation team. The alternative hypothesis (H1) is that the use of an electronic repository will improve the professional capabilities of our learners.

## 3 Materials and methods

### 3.1 Study design

**Figure 2:**
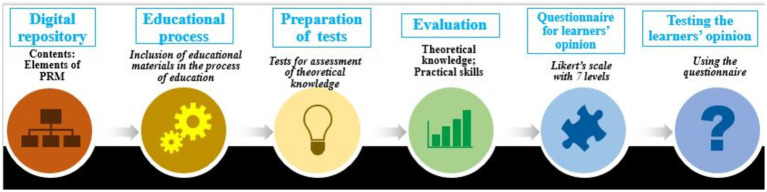
Steps in the organization of the research.

A comparative study design was used to evaluate the impact of the digitalized repository on professional competences of our students and trainees. For this, we created an electronic (e-) repository of digitized classic manuals and we prepared contemporary books, including principal elements of the European Curriculum for physical medicine and rehabilitation. These educational materials were implemented in the process of education of students, PRM trainees, and in the long-life learning programs for the members of the rehabilitation team (physicians specializing in PRM, physiatrists, physiotherapists, nurses, midwives, and medical assistants). We developed tests to assess learners’ level of theoretical knowledge. For practical skills assessment, quizzes based on clinical case scenarios were included. Finally, we conducted face-to-face both theoretical and practical exams, using the standard grading scale in our country (from Excellent 6 to Poor 2). After the exams, but before sharing their results, our students and trainees completed questionnaires for assessment of their opinions on the educational materials of the e-library and the realized educational course (lectures and practical exercises) – using a 7-levels Likert’s scale (from strongly positive to strongly negative judgment). This is a personal investigation (anonymous for the assessment of learners’ opinion), and includes a detailed analysis of results from fully completed studies and questionnaires (see Figure 2).

**Figure 3:**
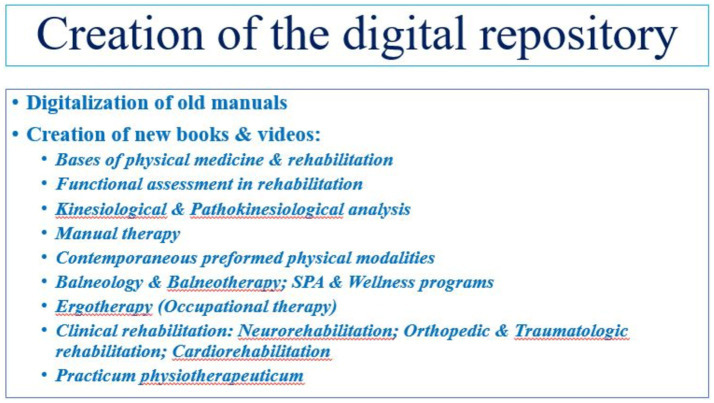
Steps in the organization of the research.

The digital repository includes e-books on different topics, related to PRM, containing theoretical data, practical issues, and case reports with videos of real patients – predominantly in Bulgarian language and some manuals in English (20–25). In the field of basic physiatry, we included books on fundamental topics: kinesiological and pathokinesiological analysis, functional evaluation; physiotherapy (active exercises, soft-tissue techniques), ergotherapy (activities), balneotherapy (mineral baths and peloids, underwater gymnastics), manual therapy (tractions, mobilizations, manipulations), preformed physical factors (electric currents, magnetic fields, ultrasound, light, laser, etc.). In the area of special physiatry, we involved manuals on: neurological and neurosurgical rehabilitation, orthopedic and traumatologic rehabilitation, rheumatologic rehabilitation, cardiac and cardiosurgical rehabilitation, robotic neurorehabilitation, etc. We added some basic publications and interesting articles on the topic (6–8, 12, 26, 27). Figure 3 clarifies the creation of the digital repository (digitalization of classic manuals and preparation of new books and videos on different topics in the area of physical medicine, physical therapy and rehabilitation).

**Figure 4:**
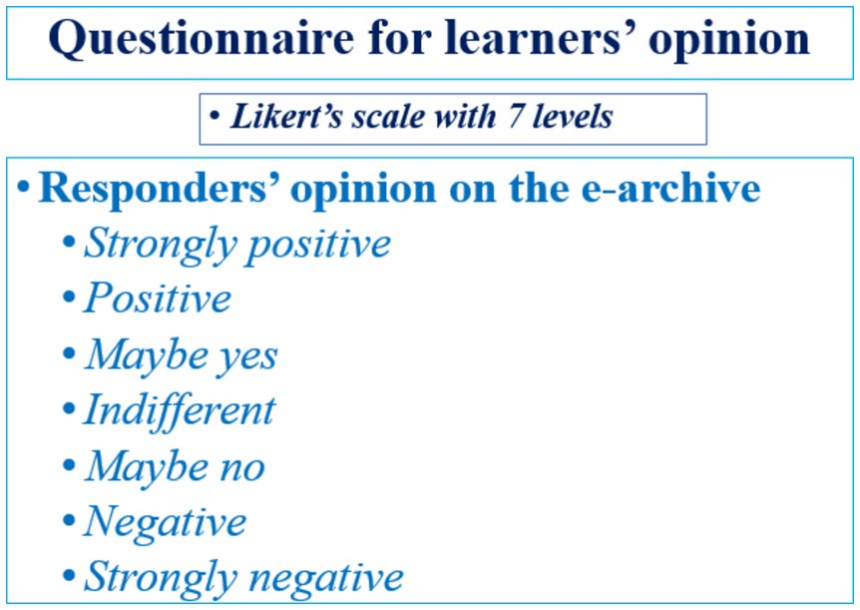
Questionnaire for learners’ opinion.

Figure 4 presents the details of the questionnaire assessing the learners’ opinion on the e-library – using a Likert’s scale with 7 levels.

### 3.2 Material

We utilized the electronic books during the education of a total of 1,186 learners: graduate studies of students in Bachelor’s degree programs (Physiotherapy, Nursing, Midwifery, Medical assistants) and Master’s degree programs (Physiotherapy, Medical rehabilitation and balneology), during the postgraduate training of medical doctors—residents in Physical and Rehabilitation medicine (PRM); and during the post-graduate long-life learning courses for physiatrists, physiotherapists, nurses, medical assistants, midwives.

#### 3.2.1 Recruitment period

Data on the application of the e-archive were collected from April 2020 to October 2024—during the education of students (Bachelor’s and Master’s degree) at the Medical University of Sofia and the Medical Faculty of Sofia University (where the first author is invited lecturer). Data for working specialists were obtained during the specialization and the state exams of residents in Physical and Rehabilitation Medicine (for obtaining the degree of PRM specialist), and during LLL-courses, organized by the Medical University of Sofia and the National Association of Healthcare professionals of Bulgaria. The results of these experimental groups were compared with the results of 787 controls. For the control groups, we used results of the exams of students and trainees from the decade before the COVID-pandemic (before the introduction of the e-archive).

#### 3.2.2 Learners’ distribution (sample size)

The material of the current study includes a total of 1973 participants, as follows:

- 401 students in Physiotherapy (PT) – Bachelor’s degree, of whom: 287 are in “Kinesitherapy” (KT) and 114 in “Medical Rehabilitation and Ergotherapy” (MRET);
- 1,166 students in Bachelor’s degree in Health care (HC) specialties – 765 in Nursing, 216 in Midwifery and 185 future Medical assistants (Med Assist);
- 171 students in Physiotherapy – Master’s degree; of them – 21 in “Kinesitherapy,” 57 in “Medical Rehabilitation and Ergotherapy” (MRET) and 93 in “Medical Rehabilitation and Balneology” (MRB);
- 30 medical doctors (MD) – residents in “Physical and Rehabilitation Medicine” (PRM);
- 205 working specialists—participants in Long-life learning (LLL) courses; of them: 161 are physiotherapists and 44 are nurses.

The responders were distributed into control groups (787 learners—traditional education and traditional exams) and experimental groups (1,186 responders – using electronic materials and applying electronic tests and face-to-face exams).

The mean age of students in Bachelor’s degree was 21 years (SD 1.6). The mean age of students in Master’s degree was 24 years (SD 1.9) The mean age of medical doctors, trainees in PRM, was 30.8 (SD 2.6). The mean age of participants in LLL-courses was 42 years (SD 7).

**Figure 5:**
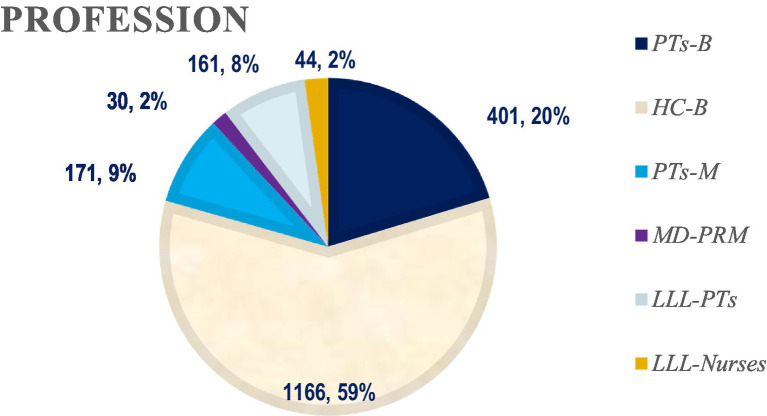
Learners’ distribution by profession (in numbers and in percent). PTs-B – Physiotherapists students in Bachelor’s degree; HC-B – Health Care professionals – students in Bachelor’s degree, PTs-M – Physiotherapists – students in Master’s degree, MD-PRM – Medical doctors – trainees in PRM, LLL-PTs – Long-life learning-physiotherapists, LLL-Nurses – Nurses during long-life learning courses.

**Figure 6:**
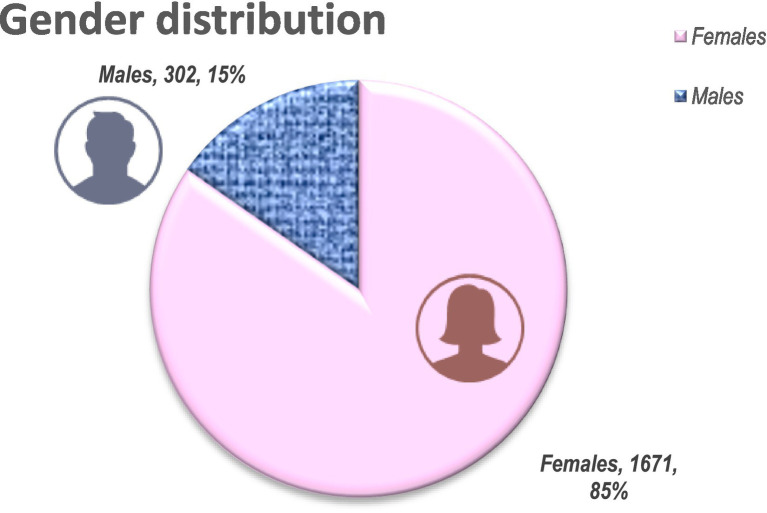
Gender distribution of participants.

We present the distribution of students and trainees by specialties (in absolute numbers and in percent) in Table 1 and Figure 5. The distribution of learners by gender (302 M: 1671 F) is presented in Figure 6.

**Table 1:** Learners’ distribution by specialty.

	Students									LLL		Total
	Bachelor’s degree					Master’s degree			MD			
	KT	MRET	Nur-sing	Midwi-fery	MedAssist	KT	MRET	MRB	PRM	PTs	Nurses	
Controls	*122*	*68*	*268*	*97*	*87*	*-*	*35*	*42*	*18*	*34*	*16*	*787*
E-learning	**165**	**46**	**497**	**119**	**98**	**21**	**22**	**51**	**12**	**127**	**28**	**1186**
	287	114	765	216	185	21	57	93	30	161	44	**1973**
	401		1166			171			30	205		**1973**
	1567					171			235			**1973**

### 3.3 Tools of data collection

#### 3.3.1 Study sites

The study was conducted at the Medical University of Sofia, Faculty of Public Health (graduate and post-graduate education of students and trainees), at several University Hospitals (during the specialization in Physical and Rehabilitation Medicine for medical doctors) and at the Bulgarian Association of Healthcare professionals (long-life learning courses for physiotherapists, nurses, midwives, and medical assistants).

#### 3.3.2 Recruitment of participants, inclusion and exclusion criteria

The enrollment of participants occurred at the beginning of the educational courses in Physical medicine, Physical therapy, Rehabilitation, Kinesiological analysis, Balneotherapy, SPA and Wellness, Manual therapy, Laser-therapy, Neurorehabilitation (NR), Orthopedic rehabilitation, Cardiorehabilitation, Grasp and gait rehabilitation, Robotic NR, etc. All students and trainees were included. We excluded learners who could not complete the respective educational course (due to personal issues). For interpreting learners’ opinion, we considered only fully completed questionnaires.

#### 3.3.3 Time schedule, intervention and outcomes

The intervention included the integration of e-learning elements during education and the use of the electronic library, sent via e-mail to all participants (with a free access link). The primary outcome was assessed at the beginning of the respective learning course, and the secondary outcome was assessed at the end of the course. Anonymous questionnaires for learners’ opinion were sent to all participants (via an Internet link).

#### 3.3.4 Follow-up and data collection

At the end of every educational course, we collected data from the practical and theoretical exams. The data from the learners’ opinion questionnaires were received after the end of the educational module, but before sharing the results of the exams.

### 3.4 Evaluation methods

For the current study, we applied different methods: screening, questionnaires, analysis of documents, and statistics.

A bank with approximately 1,000 questions for evaluation of professional competences in the field of rehabilitation was created. Different groups of tests are oriented to different types of staff in rehabilitation field (students and workers): medical doctors – PRM specialists and residents, students and health professionals in Physiotherapy (Kinesitherapy, Medical Rehabilitation and Ergotherapy, Medical rehabilitation and Balneotherapy), Nursing, Midwifery, Medical assistants. We also conducted an exploration of students’ opinion and workers’ opinion on some organization problems of Bulgarian rehabilitation school and practice.

The questionnaire was proposed to the cited target groups. Here we refer only to the results of the learners’ opinions from fully answered questionnaires (1,052 responders of a total of 1,186 participants in the experimental groups).

### 3.5 Statistical methods

The final statistical evaluation of results was conducted using the statistical package SPSS, version 19: with options for comparing two samples using parametrical analysis of variances ANOVA and non-parametrical distribution and correlation analysis, as follows: t-test (t-criterion, *p* value), Signed test, Signed rank test, Kolmogorov – Smirnov test, Mann – Whitney (Wilcoxon) W test (W median). For statistically significant effects, we considered results with a value of *p* < 0.01.

## 4 Results and analysis

The comparative analysis of the results demonstrated significant qualitative and quantitative improvement in the theoretical knowledge and practical skills of students and trainees, using the digital repository, as objectivized during online tests and face-to-face exams (theoretical and practical).

The complete evaluation is performed during theoretical and practical exams – during the final exam for physiotherapists and for medical doctors, and at the end of every educational course for specialists in the Healthcare area (nurses, midwives, medical assistants). The maximal result of the tests is 100 points. The notes of the exams in our country ranges from Poor (2) to Excellent (6).

### 4.1 Evaluation of theoretical knowledge in the basic areas of rehabilitation

**Figure 7:**
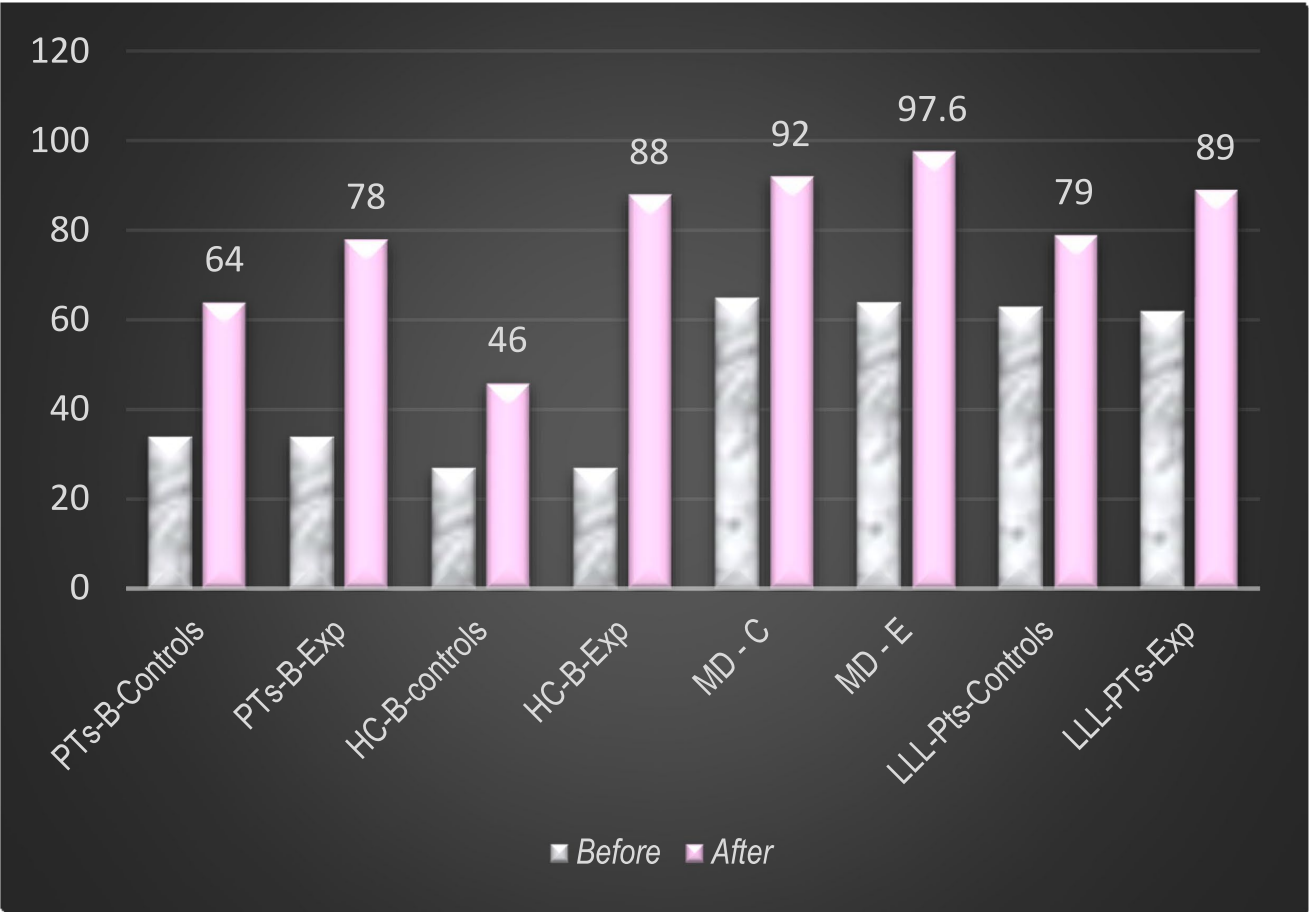
Evaluation of competencies in the basic domains of rehabilitation (maximum 100 points).

Figure 7 presents the distribution of grades (evaluation of principal bases of rehabilitation—theoretical knowledge and practical skills) among different team members: Bachelor’s and Master’s students in Physiotherapy, those in Healthcare, and working specialists during LLL-courses; with a comparison between control /C/ and experimental groups /E/.

The difference between control groups (traditional education) and experimental groups (Exp) is significant.

### 4.2 Assessment of competencies in the domain of special rehabilitation

**Figure 8:**
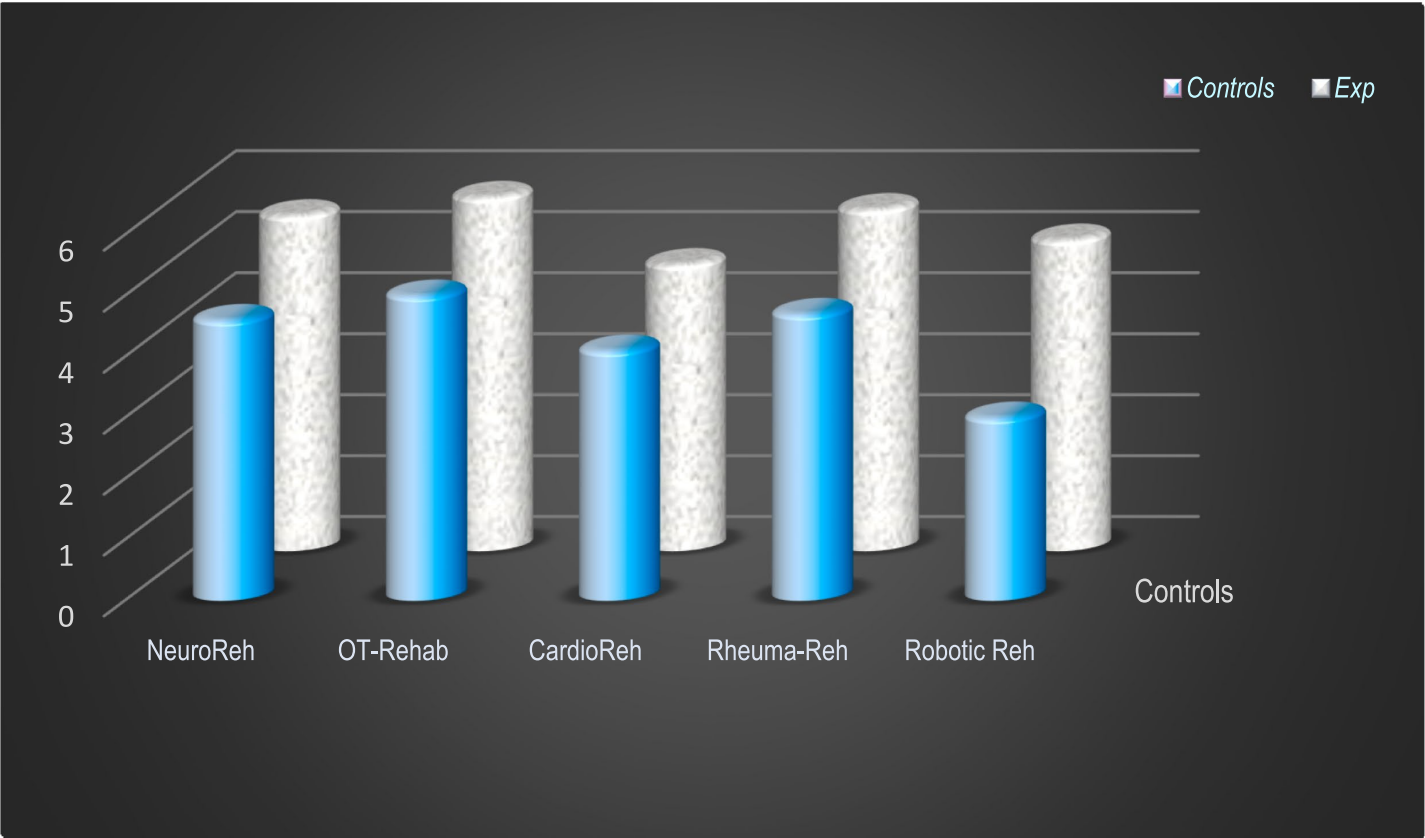
Evaluation of competencies in the special domains of rehabilitation (notes 2–6).

Next, Figure 8 presents the results of the evaluation of competencies in the fields of special rehabilitation (Reh/Rehab): neurological and neurosurgical (Neuro-Reh), orthopedic and traumatologic (OT-Rehab), cardiac and cardiosurgical (Cardio-Reh), and rheumatological rehabilitation (Rheuma-Reh). Here, we present the data of students in Master’s degree, medical doctors, and physiotherapists of LLL-courses. The comparative analysis of results from control and experimental (Exp) groups demonstrated significant differences.

### 4.3 Analysis of case studies (quizzes)

**Figure 9:**
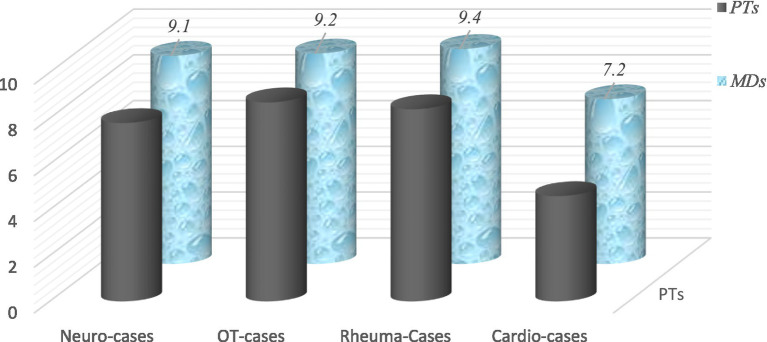
Analysis of case studies—evaluation of competencies of participants in LLL-courses (MD-PRM and PTs) (quizzes with maximal result of 10 points).

The analysis of case studies is significantly better in groups of postgraduate education and LLL-courses: medical doctors – trainees in PRM and physiotherapists. Results are presented in Figure 9. In this type of evaluation we use quizzes – clinical cases with maximal results of 10 points.

The theoretical knowledge and practical skills of medical doctors and physiotherapists are considerably better in the area of rehabilitation of neurological and orthopedic conditions. These are the most frequent patient populations and, consequently, receive the best treatment. Cardiac and cardio-surgical rehabilitation is a specific field, not sufficiently covered during university courses.

### 4.4 Analysis of learners’ opinion

**Figure 10:**
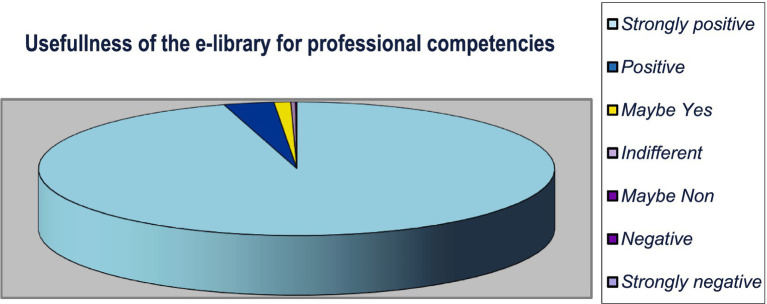
Learners’ opinion: effectiveness of the digitalized library for professional competencies.

We analyzed the learners’ opinion on the quality of the digitalized repository and its efficacy for the level of professional competences using a Likert type scale with seven echelons (from *Strongly agree* to *Strongly disagree*). Table 2 and Figure 10 present the obtained results from the fully completed questionnaires of 1,052 responders from the experimental groups.

**Table 2:** Learners’ opinion: responses from 1,052 participants in the experimental groups.

Anonymous opinion	Number of responders	Percent
Strongly positive	1,004	95.44%
Positive	33	3,14%
Maybe yes	11	1.05%
Indifferent	3	0.28%
Maybe no	1	0.000950%
Negative	0	0%
Strongly negative	0	0%
Total	1,052	100%

Practically more than 99.6% of learners expressed satisfaction with the electronic repository (the responses of 1,004 participants or 95.44% are strongly positive; 33 or 3.14% have a positive opinion, Maybe yes is the answer of 11 or 1.05%). Three participants are indifferent (0.28%). One answer is Maybe No (0.000950%). There are no negative or strongly negative opinions.

**Figure 11:**
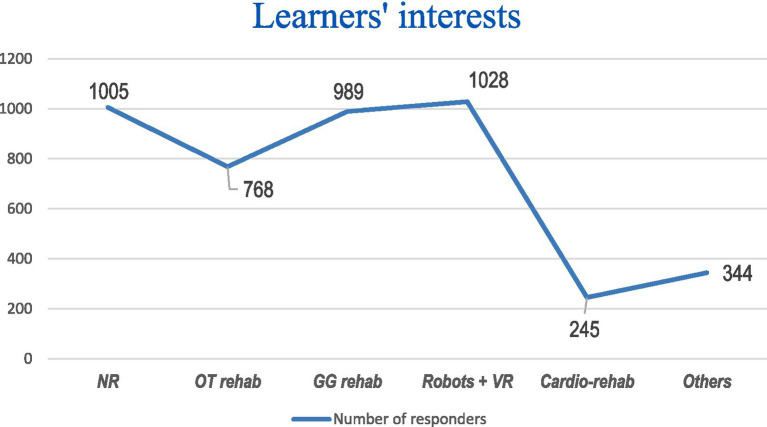
Learners’ interests. NR – Neurorehabilitation, OT rehab – Orthopedic-traumatologic rehabilitation; GG rehab – Grasp and Gait rehabilitation; VR – Virtual reality; Cardio-rehab – Cardiorehabilitation.

We asked our students and trainees about possible topics that are interesting to them. Figure 11 demonstrates the paths for amplification of the e-repository. The most interesting themes for our learners include: neurorehabilitation, neurorobots and virtual reality, grasp and gait rehabilitation, etc.

## 5 Discussion

Our findings indicate a significant enhancement in the professional competencies of learners, particularly among those in the experimental groups. The observed improvement in theoretical knowledge across fundamental domains of rehabilitation can be attributed to the implementation and utilization of the digitized repository. Notably, some participants in the experimental groups, including medical doctors, achieved scores as high as 97.6 out of a possible 100 points, reflecting an excellent level of theoretical proficiency. The development of practical skills appears to be influenced by the inclusion of practice-oriented components within the e-library, such as algorithms for functional assessment and rehabilitation, patient videos, and clinical scenarios.

In specialized areas of rehabilitation, maximal outcomes were observed in neurological, orthopedic, and rheumatological rehabilitation. These domains are particularly engaging for students and trainees, likely due to the high prevalence of related conditions in our country. A similar rationale explains the high performance observed in quiz assessments involving case studies.

All learners recognized the e-repository’s content as highly beneficial. Their interest spanned all areas of rehabilitation, with a particular emphasis on neurorehabilitation, rehabilitation of grasp and gait functions, and the integration of robotics and virtual reality in rehabilitation practices.

The content of our e-library encompasses key areas of the curriculum relevant to members of the multi-professional rehabilitation team, including medical doctors (specialists and residents in Physical and Rehabilitation Medicine), physiotherapists, nurses, and patients. The implementation of a digitalized repository has demonstrated numerous benefits, such as enhancing core professional competencies in rehabilitation and fostering the digital, intercultural, and learning skills of students and trainees (13–15, 28, 29).

Free access to educational materials promotes active learning by facilitating spaced repetition and repeated retrieval, thereby enhancing the transfer of knowledge to long-term memory. The inclusion of real-life clinical scenarios, such as video-based case studies, encourages active learner participation and emotional engagement in the educational process. Assignments involving clinical case resolution further support the practical application of theoretical knowledge, nurturing critical thinking, clinical reasoning, and decision-making skills. Despite these advantages, the role of tutors remains essential for cultivating clinical attitudes among learners. Interaction between learners and educators provides valuable feedback for both trainees and trainers. This feedback not only aids in the professional development of trainees but also inspires educators to adopt innovative teaching strategies that bridge medical theory and clinical rehabilitation practice.

Additionally, the development of digital repositories holds significant potential for advancing the integration of artificial intelligence (AI) into medicine and healthcare. For example, AI could be utilized to create question banks or design clinical cases aligned with specific learning objectives.

The comparison between traditional and electronic education (based on an e-repository) demonstrates the advantages of e-learning for the level of theoretical knowledge and practical skills of all members of the rehabilitation team. The application of the e-archive is convenient for the advancement of cognitive elements of professional competences among our students and trainees. The principal *advantages of the e-repository* include rapid access to information on new physical modalities and contemporary rehabilitation methods, resulting in the improvement of the quality of care and the quality of life of our patients (30).

Our study has some *limitations*. Currently, we have introduced only basic principles of applying robots and virtual reality in neurorehabilitation practice – for patients with post-stroke hemiplegia and in paraplegics after traumatic spinal cord injury, because we have practical experience only in this field (31). In the future, we must include in education (and, consequently, in assessment) other competencies, related to professional behavior, such as attitude, humanity, personal values, responsibility, etc. These represent possible directions for *upcoming research.*

Bulgarian students are interested in electronic education (e-platforms and training). They evaluate its advantages, as information sources from other countries, research results, the potential for information exchange and innovations, and knowledge of international standards in the field of education and rehabilitation. They consider this information crucial for the development of rehabilitative care in our country.

## 6 Conclusion and recommendations

The authors consider useful the application of electronic libraries with books, manuals, video-presentations and case studies in the graduate and post-graduate education in the rehabilitation field.

Our results demonstrate the advantages of e-learning, especially for the rapid translation of medical research and innovations.

Bulgarian learners consider the electronic repository useful.

The modernization of didactic concepts should promote education in the rehabilitation field and adapt education to the needs of clinical practice, ultimately improving the quality of patient care.

We recommend the application of digitalized repositories in the process of education of students and trainees.

## Data Availability

Requests to access the datasets should be directed to dr.yvette.5@gmail.com.
